# Comparative analysis of two-dimensional and three-dimensional machine learning models for predicting the degree of invasion in lung non-mucinous adenocarcinomas presenting as subsolid nodules

**DOI:** 10.3389/fonc.2026.1916349

**Published:** 2026-09-16

**Authors:** Yi-Ming Hui, Ming-Zhi Lin, Bin Li, Yu-Qi Meng, Hai-Ming Feng, Yu-Zhen Chen, Zhi-Peng Su

**Affiliations:** Department of Thoracic Surgery, The Second Hospital and Clinical Medical School, Lanzhou University, Lanzhou, Gansu, China

**Keywords:** consolidation-to-tumor ratio of volume, lung adenocarcinoma, machine learning, subsolid nodules, three-dimensional

## Abstract

**Introduction:**

Most malignant subsolid nodules (SSNs) are non-mucinous adenocarcinomas, and prognosis varies substantially with the degree of invasion. Previous studies have shown that the solid component of SSNs is closely associated with invasiveness. With advances in image analysis, three-dimensional (3D) computed tomography (CT) features may provide more comprehensive information than conventional two-dimensional (2D) measurements. This study aimed to investigate the value of 2D and 3D CT features for predicting the extent of invasion in SSNs and to compare the diagnostic performance and potential clinical utility of 2D- and 3D-based predictive models.

**Materials and methods:**

This single-center retrospective model-development study included 234 SSNs from 223 patients with pathologically confirmed lung non-mucinous adenocarcinoma. Minimally invasive adenocarcinoma and Grade 1 tumors were classified as the low-risk group, whereas Grades 2 and 3 tumors were classified as the high-risk group. Patients were randomly assigned at the patient level to a training set comprising 171 SSNs from 164 patients and a held-out internal test set comprising 63 SSNs from 59 patients. 2D and 3D CT parameters, including consolidation-to-tumor ratio of volume (CTRV), were evaluated. Least absolute shrinkage and selection operator regression was used for feature selection, and models based on five machine-learning algorithms were developed using 5×5-fold nested cross-validation within the training set. The best-performing 2D and 3D models were selected according to the area under the receiver operating characteristic curve (AUC) calculated from pooled out-of-fold (OOF) predictions and were subsequently evaluated in the held-out test set. Model performance was assessed in terms of discrimination, calibration, and decision curve analysis, and AUCs were compared using the paired DeLong test.

**Results:**

The best-performing 2D and 3D models were 2D-XGB and 3D-RF, respectively, with pooled OOF AUCs of 0.713 (0.632–0.790) and 0.712 (0.633–0.785). In the held-out test set, 2D-XGB achieved an AUC of 0.766 (0.648–0.869), whereas 3D-RF achieved an AUC of 0.847 (0.745–0.931). No significant difference in AUC was observed between the two models based on either the pooled OOF predictions or the held-out test set predictions. Permutation importance analysis indicated that consolidation-to-tumor ratio was the most influential feature in 2D-XGB, whereas mass and CTRV_-100_ were among the most important features in 3D-RF. Compared with 2D-XGB, 3D-RF showed higher net benefit across a wider range of threshold probabilities and better calibration.

**Conclusions:**

Both 2D- and 3D-based models showed comparable discriminative performance for predicting the degree of invasion in lung non-mucinous adenocarcinomas presenting as SSNs. The 3D-RF model showed better calibration and a broader range of clinical net benefit, suggesting potential advantages for risk estimation and clinical decision-making. Mass and CTRV derived from multiple density thresholds may provide additional information for characterizing solid components and may support clinical decision-making.

## Introduction

Pulmonary nodules are defined as focal opacities measuring up to 3 cm in diameter and surrounded by lung parenchyma, including nodules adjacent to the pleura. On chest computed tomography (CT), pulmonary nodules are classified according to density as solid nodules or subsolid nodules (SSNs), with SSNs further categorized as pure ground-glass nodules or part-solid nodules (PSNs) (1). With the implementation and widespread adoption of low-dose computed tomography for lung cancer screening, increasing numbers of pulmonary nodules, particularly early-stage and indeterminate lesions, are being detected (2). Pulmonary nodules may be benign or malignant. Non-mucinous adenocarcinoma is the most common malignancy presenting as an SSN. According to the 2021 WHO classification of lung tumors, non-mucinous adenocarcinomas are further classified as non-mucinous minimally invasive adenocarcinoma (MIA), Grade 1 (well-differentiated, lepidic-predominant tumors with no or <20% high-grade pattern), Grade 2 (moderately differentiated, acinar- or papillary-predominant tumors with no or <20% high-grade pattern), or Grade 3 (poorly differentiated tumors with ≥20% high-grade pattern; high-grade patterns include micropapillary, solid, cribriform, and complex glandular patterns) (3). The five-year recurrence-free survival of patients with MIA and Grade 1 tumors is close to 100%, whereas it is significantly lower for those with Grade 2 or 3 tumors (4–6).

Several studies have demonstrated that the solid component of SSNs is closely associated with the degree of invasion. However, most previous studies have relied on two-dimensional (2D) parameters, such as the size of the solid component or the consolidation-to-tumor ratio (CTR) (7–10). Although 2D parameters are widely used in clinical practice and have been adopted as indicators for surgical decision-making (11, 12), pulmonary nodules and their solid components may have irregular shapes, limiting the ability of 2D measurements to fully characterize their spatial extent. Advances in image analysis and artificial intelligence have enabled more accurate fully or semi-automated quantification of three-dimensional (3D) nodule parameters (13–15). These 3D parameters may provide additional information beyond conventional 2D measurements. Common 3D parameters include volume, mass, mean CT attenuation, consolidation-to-tumor ratio of volume (CTRV), and volume of the solid component (VSC). Previous studies have demonstrated the utility of CTRV using different approaches to defining the solid component, including manual measurement and CT attenuation thresholds ≥-300 HU and -250 HU, together with different grouping strategies, potentially reflecting differences in study objectives (14, 16, 17). However, no clear guidelines or expert consensus currently exist regarding the optimal density threshold for distinguishing ground-glass from solid components when calculating CTRV and VSC.

This study aimed to investigate the value of 2D and 3D CT features, including solid components defined using different density thresholds, for predicting the degree of invasion in lung non-mucinous adenocarcinomas presenting as SSNs, and to compare the diagnostic performance and potential clinical utility of prediction models based on 2D and 3D features.

## Materials and methods

### Study population and patient selection

This single-center retrospective model-development study included 234 SSNs from 223 patients with pathologically confirmed lung non-mucinous adenocarcinoma who underwent evaluation between January 2020 and January 2024. Of the 234 SSNs, 54 were classified as minimally invasive adenocarcinoma (MIA), 75 as Grade 1, 90 as Grade 2, and 15 as Grade 3. MIA and Grade 1 tumors were categorized as the low-risk group, whereas Grade 2 and Grade 3 tumors were categorized as the high-risk group. The study protocol was approved by the Medical Ethics Committee (approval No. 2024A-133; February 23, 2024), and the requirement for written informed consent was waived because of the retrospective study design. The inclusion criteria were as follows: (a) an SSN identified on chest CT performed within 1 month before surgery; and (b) complete surgical records documenting pulmonary nodule resection, with a pathologic diagnosis of lung non-mucinous adenocarcinoma according to the 2021 WHO classification of lung tumors. The exclusion criteria were as follows: (a) receipt of preoperative therapy; (b) inadequate CT image quality; and (c) a pathologic diagnosis of metastatic lung cancer. Patients were randomly assigned at the patient level to either the training set or the held-out test set. The training set comprised 164 patients with 171 SSNs, whereas the held-out test set comprised 59 patients with 63 SSNs. All nodules from a given patient were assigned exclusively to the same dataset, ensuring that no patient contributed nodules to both the training and held-out test sets. A detailed flowchart of patient enrollment is shown in **Figure 1**.

**Figure 1:**
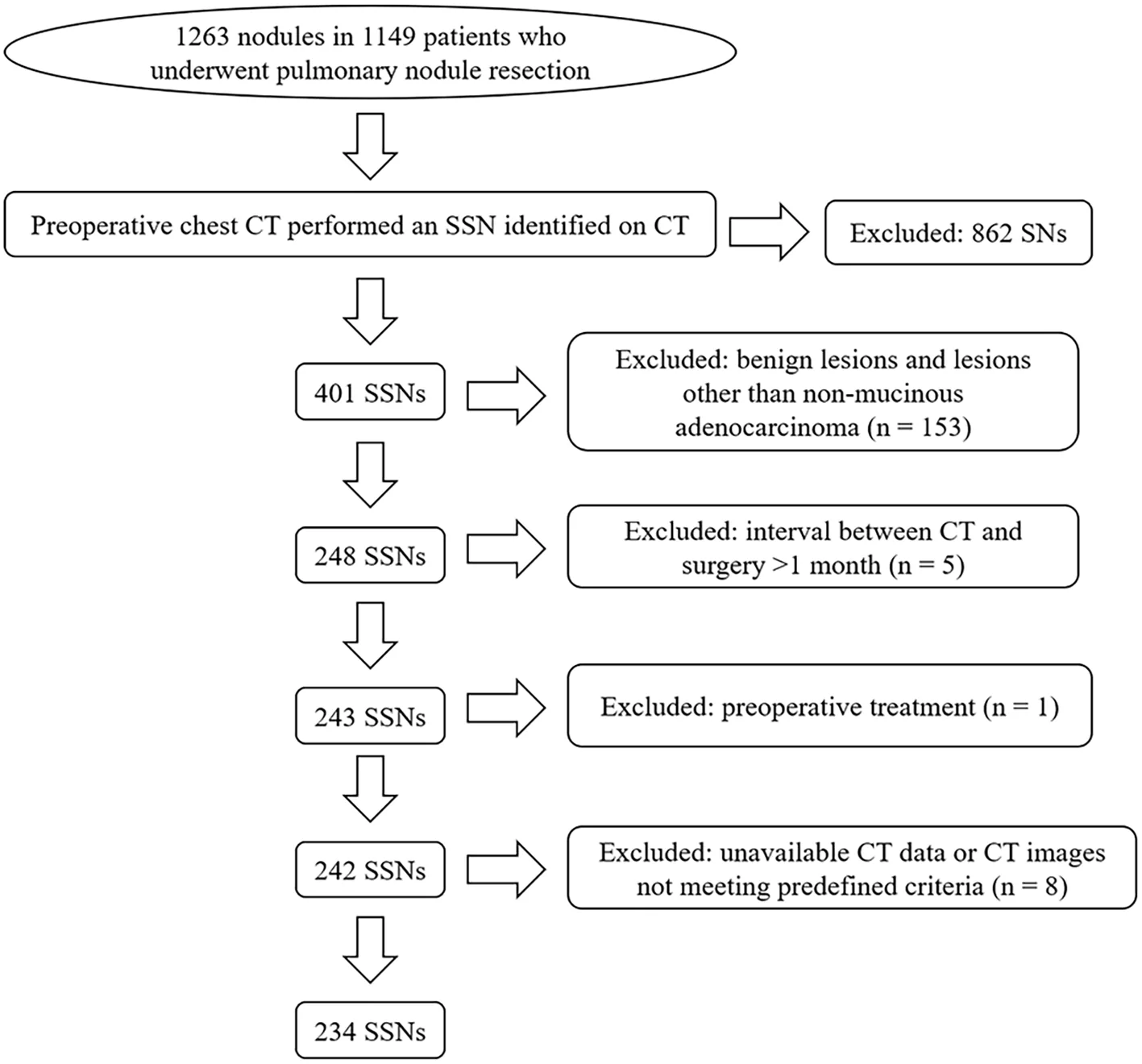
Flowchart of study population selection.

### CT acquisition and image reconstruction

CT examinations were performed using three scanners: Discovery CT750 HD (GE Healthcare), Philips iCT 256 (Koninklijke Philips N.V.), and Somatom Sensation 64 (Siemens, Erlangen, Germany). Scanning parameters were as follows: tube voltage, 120 kVp; tube current, 150–200 mA; axial section thickness and spacing, 5 mm each; and reconstruction section thickness and interval, 1.25 mm each. All CT images were evaluated using lung-window settings (width, 1500–2000 HU; level, −450 to −600 HU) and mediastinal-window settings (width, 300–350 HU; level, 30–50 HU).

### Clinical and CT feature assessment

Clinical information, including age, sex, surgical records, and pathology reports, was collected. Semantic CT features of SSNs, including location, irregularity, spiculation, lobulation, pleural indentation, vacuole, air bronchogram, and vessel convergence, were independently assessed by two radiologists who were blinded to the pathologic findings. Disagreements were resolved by a senior radiologist. The maximum size of the solid component was measured on lung-window images by both radiologists, and the mean of their measurements was used for analysis.

Chest CT images were imported into artificial intelligence (AI) software (Deepwise AI Medical Image Assisted Diagnosis System, Beijing, China) to obtain the maximum size, volume, mass, mean CT attenuation, and CTRV and VSC values at density thresholds ranging from −500 HU to −100 HU in 50-HU increments. For example, for CTRV_−300_ and VSC_−300_, voxels with attenuation values ≥−300 HU were classified as the solid component. The maximum nodule size and maximum size of the solid component were recorded as rounded integers according to the 2017 Fleischner Society guidelines (18). CTR was defined as the ratio of the maximum size of the solid component to the maximum nodule size, whereas CTRV was defined as the ratio of the volume of the solid component to the total nodule volume.

### Machine-learning model development and internal validation

Univariable analyses were first performed using the full training set, and variables showing statistically significant between-group differences were retained as candidate variables for subsequent model development. The held-out internal test set was not included in this screening procedure. Model development was performed within the training set using nested cross-validation with five outer folds and five inner folds, with identical outer-fold assignments used for the 2D and 3D analyses. For each outer fold, the held-out outer fold was reserved for out-of-fold (OOF) prediction. Within the corresponding outer training subset, five-fold inner cross-validation was used for feature selection and hyperparameter optimization. For each inner fold, candidate continuous 2D or 3D CT parameters were subjected to cross-validated least absolute shrinkage and selection operator (LASSO) regression using only the inner training data. LASSO was implemented using a binomial model with α=1 and binomial deviance as the cross-validation metric, with λ min used for variable selection. The selected continuous CT parameters were then combined with the categorical variables retained from the univariable analyses. Five prespecified machine-learning algorithms were evaluated: logistic regression (LR), support vector machine (SVM), random forest (RF), extreme gradient boosting (XGB), and Light Gradient Boosting Machine (LGBM). For each fixed algorithm, hyperparameters were optimized according to performance across the inner validation folds. After hyperparameter optimization, LASSO feature selection was repeated using the complete outer training subset, and the resulting model was fitted using the outer training data and applied to the held-out outer fold. Thus, the outer validation fold was not used for LASSO feature selection, hyperparameter optimization, or model fitting. Each of the five prespecified algorithms was evaluated separately across all five outer folds. The resulting predictions from the five held-out outer folds were pooled to calculate an OOF area under the receiver operating characteristic curve (AUC) for each fixed algorithm. The algorithm with the highest pooled OOF AUC was selected separately for the 2D and 3D feature sets. The same outer-fold assignments were used for both analyses. After selection of the best 2D and 3D algorithms, the final modeling pipeline was refitted using the complete training set. LASSO feature selection was repeated on the complete training set, and hyperparameters for the selected algorithms were optimized using five-fold cross-validation within the training set. The final 2D and 3D models were then fitted using the complete training set and frozen before evaluation in the held-out test set. The held-out test set was not used for univariable screening, LASSO feature selection, hyperparameter optimization, algorithm selection, classification-threshold determination, or any other stage of model development. Model-agnostic permutation importance was used to interpret the final 2D and 3D models. This analysis was performed only after model development had been completed and was not involved in feature selection, algorithm selection, hyperparameter optimization, or classification-threshold determination. Each predictor was randomly permuted while all other variables were kept unchanged, and the model AUC was recalculated. Permutation importance was defined as the difference between the baseline AUC and the AUC after permutation of the corresponding predictor. Each predictor was permuted 50 times, and the mean decrease in AUC was used as the measure of feature importance. The overall modeling workflow is shown in **Figure 2**.

**Figure 2:**
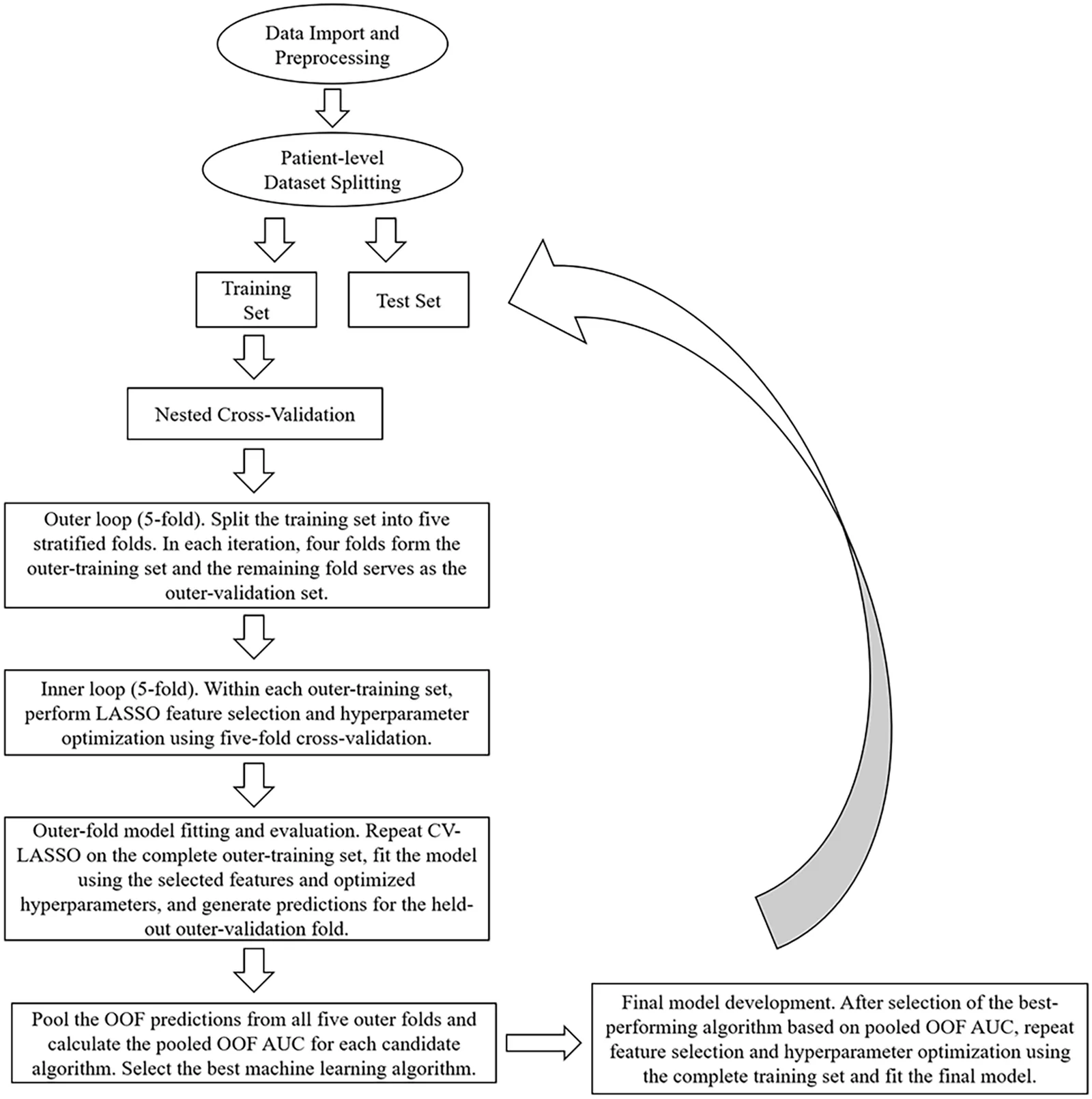
Workflow for machine-learning model development and internal validation.

### Statistical analysis

Statistical analyses were performed using SPSS version 27.0 and R version 4.6.1. Detailed information on the software packages and versions, data preprocessing and standardization, handling of missing data, hyperparameter search spaces, random-seed settings, and approaches to class imbalance is provided in **Supplementary File S1**. Random allocation to the training and held-out test sets was performed at the patient level. The intraclass correlation coefficient (ICC) was used to assess agreement between the two radiologists for measurements of the maximum size of the solid component, with an ICC >0.8 indicating good agreement. Categorical variables were compared between the low- and high-risk groups using the χ² test, continuity-corrected χ² test, or Fisher exact test, as appropriate, whereas continuous variables were compared using the Mann–Whitney U test. Spearman rank correlation analysis was used to evaluate associations between individual variables and the high-risk group. All statistical tests were two-sided, and P<0.05 was considered statistically significant. Model discrimination was assessed using the AUC. For both the pooled OOF predictions and held-out test set predictions, AUCs and their 95% confidence interval (CI) were estimated using 30,000 stratified bootstrap resamples. The AUCs of the best 2D and 3D models were compared using the paired DeLong test. Differences in AUC between the 2D and 3D models were additionally estimated using paired bootstrap 95% CIs. For each final model, the classification threshold was determined from the pooled OOF predictions using the Youden index and was subsequently applied unchanged to the held-out test set, without re-optimization in the test set. Sensitivity and specificity were calculated using these fixed thresholds, with 95% CIs estimated using the exact binomial method. Decision curve analysis (DCA) was performed in the held-out test set to assess potential clinical utility by comparing the net benefit of the models with the treat-all and treat-none strategies across threshold probabilities ranging from 0.05 to 0.95. Model calibration in the held-out test set was assessed using calibration curves, the Brier score and calibration slope.

## Results

The ICC for the maximum size of the solid component measured by the two radiologists was 0.991, indicating a high degree of agreement. In the training set, univariable analysis showed significant between-group differences in irregularity, spiculation, lobulation, pleural indentation, air bronchogram, and all CT parameters (all P<0.05); these variables were also significantly associated with the high-risk group (all P<0.05; r=0.165–0.362). In contrast, age, sex, location, vacuole, and vessel convergence showed no significant between-group differences (all P>0.05) or significant associations with the high-risk group (all P>0.05; r=0.006–0.131) (**Table 1**). In the held-out test set, age, irregularity, spiculation, air bronchogram, and all CT parameters differed significantly between the two groups (all P<0.05) and were significantly associated with the high-risk group (all P<0.05; r=0.255–0.489). Sex, location, lobulation, pleural indentation, vacuole, and vessel convergence showed no significant between-group differences (all P>0.05) or significant associations with the high-risk group (all P>0.05; r=0.004–0.142) (**Table 1**).

**Table 1:** Characteristics of low-risk and high-risk groups for the training set and test set.

	Training set					Test set				
Characteristics	Low-risk (n=92)	High-risk (n=79)	P^*^	r	P^†^	Low-risk (n=37)	High-risk (n=26)	P^*^	r	P^†^
Age	55.5 (50, 60.75)	57 (52, 64)	0.088	0.131	0.088	55 (49.5, 59)	59.5 (55.25, 64.5)	0.022	0.29	0.021
Sex			0.934	0.006	0.934			0.379	0.111	0.387
Male	32	27				13	12			
Female	60	52				24	14			
Upper lobe			0.244	0.089	0.246			0.941	0.009	0.942
Yes	67	51				21	15			
No	25	28				16	11			
Irregular			0.012	0.192	0.012			0.002	0.435	<0.001
Yes	26	37				4	13			
No	66	42				33	13			
Spiculation			0.003	0.228	0.003			0.003	0.374	0.002
Yes	20	34				9	16			
No	72	45				28	10			
Lobulation			0.031	0.165	0.031			0.518	0.081	0.526
Yes	19	28				10	9			
No	73	51				27	17			
Pleural indentation			0.003	0.23	0.002			0.564	0.073	0.571
Yes	28	42				13	11			
No	64	37				24	15			
Vacuole			0.284	0.082	0.287			0.975	0.004	0.976
Yes	15	18				7	5			
No	77	61				30	21			
Air bronchogram			<0.001	0.362	<0.001			<0.001	0.489	<0.001
Yes	48	68				21	26			
No	44	11				16	0			
Vessel convergence			0.204	0.097	0.207			0.259	0.142	0.266
Yes	47	48				16	15			
No	45	31				21	11			
Maximum size (mm)	13.5 (9, 19)	16 (12, 20)	0.003	0.224	0.003	12 (9, 18.5)	18 (13, 22.25)	0.004	0.361	0.004
Maximum size of the solid component (mm)	0 (0, 6)	7 (0, 11)	<0.001	0.295	<0.001	0 (0,4)	6 (0, 9.5)	0.006	0.347	0.005
CTR (%)	0 (0, 44.95)	45.46 (0, 60)	<0.001	0.297	<0.001	0 (0, 39.64)	40.63 (0, 57.33)	0.013	0.316	0.012
Mean CT attenuation value (HU)	-543.95 (-637.18, -441.25)	-464.7 (-557.7, -331.7)	<0.001	0.268	<0.001	-544.3 (-612.15, -440.75)	-412.05 (-582.05, -303.33)	0.044	0.255	0.043
Volume (mm^3^)	863.45 (417.69, 1914.69)	1564.02 (770.18, 2576.81)	0.001	0.252	<0.001	753.4 (380.78, 2247.69)	1598.36 (928.54, 3021.19)	0.015	0.309	0.014
Mass (mg)	335.8 (182.48, 809.88)	699 (368.2, 1306.8)	<0.001	0.301	<0.001	290.7 (148.35, 861.05)	800.95 (397.73, 1564.35)	0.005	0.355	0.004
CTRV_-500_(%)	0 (0, 33.23)	36.8 (0,60.7)	<0.001	0.301	<0.001	0 (0, 34.7)	42.15 (0, 67.45)	0.015	0.309	0.014
VSC_-500_(mm^3^)	0 (0, 264.65)	470.08 (0, 1033.73)	<0.001	0.288	<0.001	0 (0, 160.3)	507.5 (0, 1056.65)	0.013	0.317	0.011
CTRV_-450_(%)	0 (0, 25.98)	30.8 (0, 53.7)	<0.001	0.304	<0.001	0 (0, 28.6)	35.95 (0, 60.48)	0.012	0.319	0.011
VSC_-450_(mm^3^)	0 (0, 226.5)	413.42 (0, 959.7)	<0.001	0.292	<0.001	0 (0, 130.53)	415.74 (0, 912.09)	0.013	0.315	0.012
CTRV_-400_(%)	0 (0, 20.58)	25.8 (0, 47.8)	<0.001	0.309	<0.001	0 (0, 23.7)	29.9 (0, 53.18)	0.011	0.323	0.01
VSC-_400_(mm^3^)	0 (0,197.89)	352.51 (0, 901.94)	<0.001	0.292	<0.001	0 (0, 98.35)	342.91 (0, 792.46)	0.012	0.319	0.011
CTRV_-350_(%)	0 (0,16.8)	22.5 (0, 41.1)	<0.001	0.314	<0.001	0 (0, 17.65)	24.95 (0, 44.63)	0.009	0.333	0.008
VSC-_350_(mm^3^)	0 (0, 163.55)	291.55 (0, 787.5)	<0.001	0.295	<0.001	0 (0, 66.99)	279.62 (0, 687.6)	0.011	0.324	0.01
CTRV_-300_(%)	0 (0, 14.55)	18.3 (0,37)	<0.001	0.318	<0.001	0 (0, 13.2)	20.55 (0, 38.15)	0.007	0.345	0.006
VSC_-300_(mm^3^)	0 (0, 121.1)	257.14 (0, 666.86)	<0.001	0.298	<0.001	0 (0, 46.78)	223.63 (0, 595.92)	0.011	0.322	0.01
CTRV_-250_(%)	0 (0, 12.38)	13.8 (0, 30.6)	<0.001	0.319	<0.001	0 (0, 9.85)	16.6 (0, 32.93)	0.005	0.353	0.005
VSC-_250_(mm^3^)	0 (0, 102.33)	193.12 (0, 561.86)	<0.001	0.298	<0.001	0 (0, 29.05)	181.21 (0, 522.05)	0.009	0.33	0.008
CTRV_-200_(%)	0 (0, 9.18)	10.6 (0, 25.8)	<0.001	0.32	<0.001	0 (0, 7.4)	12.7 (0, 27.05)	0.004	0.361	0.004
VSC_-200_(mm^3^)	0 (0, 80.4)	146.64 (0, 457.98)	<0.001	0.297	<0.001	0 (0, 20.64)	139.71 (0, 439.76)	0.009	0.332	0.008
CTRV_-150_(%)	0 (0, 6.83)	8.1 (0, 21.7)	<0.001	0.322	<0.001	0 (0, 4.95)	9.25 (0, 20.75)	0.003	0.372	0.003
VSC_-150_(mm^3^)	0 (0, 62.63)	107.16 (0, 401.43)	<0.001	0.298	<0.001	0 (0, 15.63)	99.76 (0, 375.37)	0.008	0.338	0.007
CTRV_-100_(%)	0 (0, 5.58)	6.6 (0, 18.1)	<0.001	0.325	<0.001	0 (0, 3.8)	7.5 (0, 15.78)	0.003	0.376	0.002
VSC-_100_(mm^3^)	0 (0, 54.6)	75.35 (0, 365.63)	<0.001	0.301	<0.001	0 (0, 10.73)	68.84 (0, 315.39)	0.007	0.343	0.006

CTR, consolidation-to-tumor ratio; CTRV, consolidation-to-tumor ratio of volume at different thresholds; VSC, volume of the solid component at different thresholds. ^*^Intergroup comparison of low-risk and high-risk groups; ^†^The Spearman’s correlation analyses of low-risk and high-risk groups.

The LASSO coefficient-path and cross-validation plots for the 2D and 3D analyses are shown in **Supplementary Figure 1**. The pooled OOF AUCs of 2D-LR, 2D-SVM, 2D-RF, 2D-XGB, and 2D-LGBM were 0.682 (0.602–0.759), 0.666 (0.583–0.745), 0.707 (0.627–0.783), 0.713 (0.632–0.790), and 0.677 (0.595–0.755), respectively. Based on the highest pooled OOF AUC, XGB was selected as the final 2D algorithm (**Figure 3A**; **Table 2**). The optimized hyperparameters for the final 2D-XGB model were max_depth=2, learning_rate=0.05, and nrounds=50. The pooled OOF AUCs of 3D-LR, 3D-SVM, 3D-RF, 3D-XGB, and 3D-LGBM were 0.687 (0.605–0.765), 0.684 (0.601–0.763), 0.712 (0.633–0.785), 0.689 (0.608–0.765), and 0.679 (0.598–0.759), respectively. Based on the highest pooled OOF AUC, RF was selected as the final 3D algorithm (**Figure 3B**; **Table 2**). The optimized hyperparameters for the final 3D-RF model were mtry=1 and nodesize=5, with the number of trees fixed at 500.

**Figure 3:**
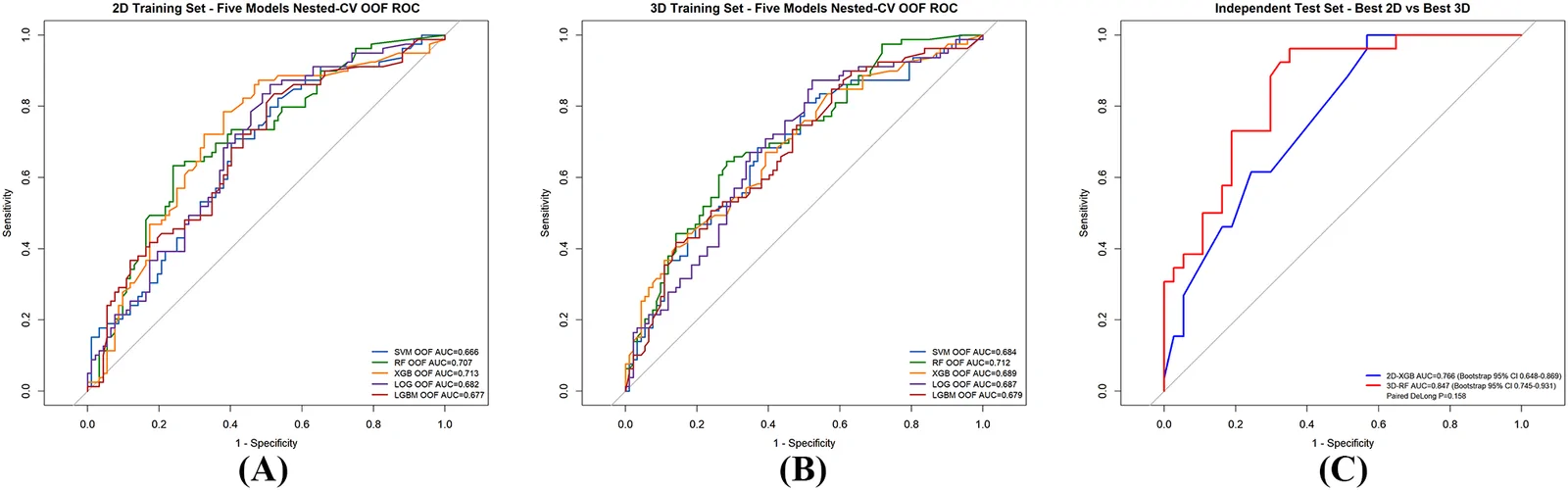
Receiver operating characteristic curves of the 2D and 3D machine-learning models in nested cross-validation and the held-out test set. **(A)** ROC curves of the five 2D machine-learning algorithms based on pooled OOF predictions generated by nested cross-validation in the training set. **(B)** ROC curves of the five 3D machine-learning algorithms based on pooled OOF predictions generated by nested cross-validation in the training set. **(C)** ROC curves of the final 2D-XGB and 3D-RF models in the held-out test set (independent test set). The best-performing 2D and 3D algorithms were selected according to their pooled OOF AUC in the training set. AUC, area under the receiver operating characteristic curve; LGBM, Light Gradient Boosting Machine; LR, logistic regression; OOF, out-of-fold; RF, random forest; ROC, receiver operating characteristic; SVM, support vector machine; XGB, extreme gradient boosting.

**Table 2:** Discriminative performance of the 2D and 3D machine-learning models based on nested cross-validation in the training set.

Model	Pooled OOF AUC (95% CI)	Mean outer-fold AUC	SD of outer-fold AUC	OOF-derived cutoff	OOF sensitivity (95% CI)	OOF specificity (95% CI)
2D-LR	0.682 (0.602-0.759)	0.697	0.056	0.381	0.861 (0.765-0.928)	0.489 (0.383-0.596)
2D-SVM	0.666 (0.583-0.745)	0.683	0.070	0.455	0.709 (0.596-0.806)	0.587 (0.479-0.689)
2D-RF	0.707 (0.627-0.783)	0.715	0.066	0.521	0.633 (0.517-0.739)	0.761 (0.661-0.844)
2D-XGB	0.713 (0.632-0.790)	0.702	0.068	0.393	0.785 (0.678-0.869)	0.620 (0.512-0.719)
2D-LGBM	0.677 (0.595-0.755)	0.688	0.069	0.339	0.835 (0.735-0.909)	0.478 (0.373-0.585)
3D-LR	0.687 (0.605-0.765)	0.698	0.073	0.354	0.873 (0.780-0.938)	0.478 (0.373-0.585)
3D-SVM	0.684 (0.601-0.763)	0.704	0.074	0.442	0.684 (0.569-0.784)	0.630 (0.523-0.729)
3D-RF	0.712 (0.633-0.785)	0.720	0.059	0.475	0.646 (0.530-0.750)	0.717 (0.614-0.806)
3D-XGB	0.689 (0.608-0.765)	0.690	0.060	0.461	0.671 (0.556-0.773)	0.609 (0.501-0.709)
3D-LGBM	0.679 (0.598-0.759)	0.683	0.075	0.604	0.418 (0.308-0.534)	0.859 (0.770-0.923)

Pooled OOF AUC was calculated from the pooled out-of-fold predictions generated across the five outer folds of nested cross-validation. Mean outer-fold AUC represents the mean AUC across the five outer-validation folds, and SD of outer-fold AUC represents the corresponding standard deviation. The OOF-derived cutoff was determined from the pooled OOF predictions and was used to calculate OOF sensitivity and specificity. The best-performing algorithm for each feature set was selected according to the pooled OOF AUC. OOF, out-of-fold; AUC, area under the receiver operating characteristic curve; CI, confidence interval; LGBM, Light Gradient Boosting Machine; LR, logistic regression; RF, random forest; SD, standard deviation; SVM, support vector machine; XGB, extreme gradient boosting.

Following LASSO feature selection, the 2D parameters included in the final 2D-XGB model were maximum size, maximum size of the solid component, and CTR, with fold-wise selection frequencies of 0.4, 0.8, and 1.0, respectively. The 3D parameters included in the final 3D-RF model were mass, CTRV_-100_, CTRV_-500_ and CTRV_-300_, with fold-wise selection frequencies of 0.6, 0.6, 0.4, and 0.4, respectively. Permutation importance analysis showed that CTR, maximum size, and air bronchogram were among the most important features in the 2D-XGB model, whereas mass, CTRV_-100_ and air bronchogram were among the most important features in the 3D-RF model (**Figure 4**). Based on the pooled OOF predictions, the difference in AUC between the 2D-XGB and 3D-RF models was −0.001 (95% CI, −0.069 to 0.065), and the paired DeLong test showed no statistically significant difference (P = 0.960). In the held-out test set, the AUC was 0.766 (0.648–0.869) for the 2D-XGB model and 0.847 (0.745–0.931) for the 3D-RF model. The difference in AUC between the two models was 0.081 (95% CI, −0.029 to 0.195), and the paired DeLong test showed no statistically significant difference (P = 0.158) (**Figure 3C**; **Table 3**). DCA in the held-out test set showed that both 2D-XGB and 3D-RF provided net benefit across nearly the entire range of evaluated threshold probabilities. The net benefit of 3D-RF was higher than that of 2D-XGB over most of the threshold probability range, particularly at intermediate-to-high threshold probabilities (**Figure 5**). In the held-out test set, the 2D-XGB model had a Brier score of 0.196 and a calibration slope of 1.73. The calibration curve of the 3D-RF model was generally closer to the ideal reference line, with a Brier score of 0.162 and a calibration slope of 1.15 (**Figure 6**).

**Figure 4:**
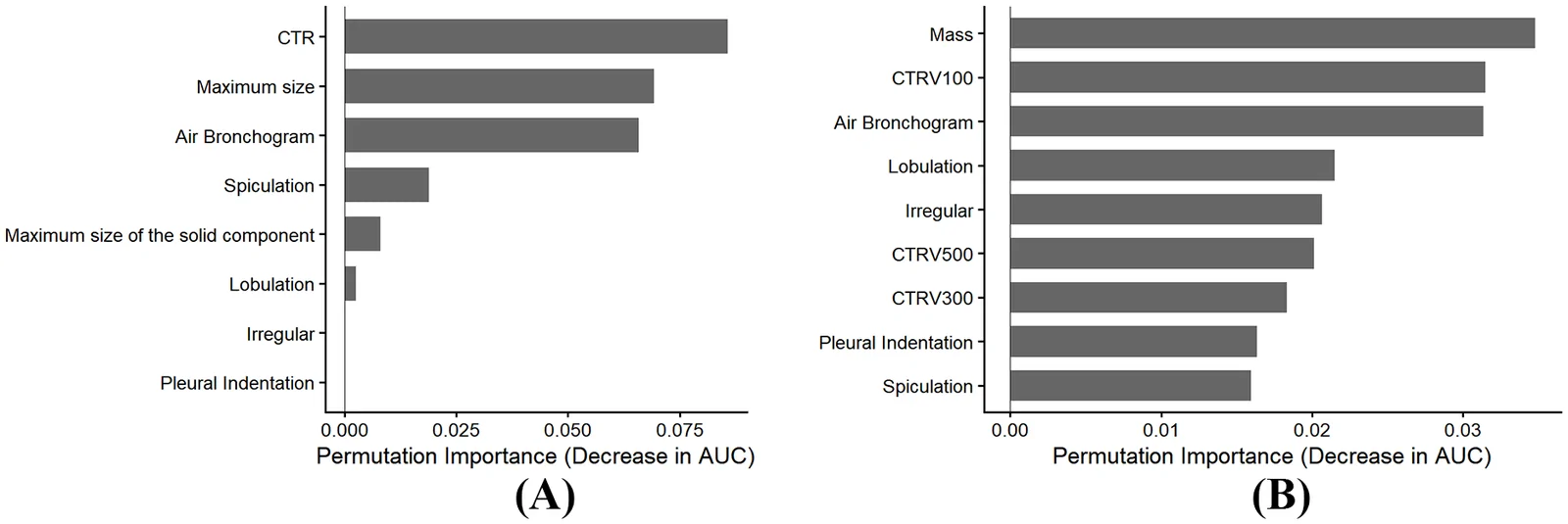
Permutation feature importance of the final 2D-XGB and 3D-RF models in the held-out test set. **(A)** Permutation feature importance of the final 2D-XGB model. **(B)** Permutation feature importance of the final 3D-RF model. Feature importance was quantified as the decrease in the AUC after permutation of each feature in the held-out test set, with larger decreases indicating greater contributions to model discrimination. Error bars represent the variability in AUC decrease across repeated permutations. AUC, area under the receiver operating characteristic curve; CTR, consolidation-to-tumor ratio; RF, random forest; VSC, volume of the solid component; CTRV, consolidation-to-tumor ratio of volume; XGB, extreme gradient boosting.

**Table 3:** Performance of the final 2D-XGB and 3D-RF models in the held-out test set.

Model	LASSO-selected quantitative features	Test AUC (95% CI)	Test sensitivity (95% CI)	Test specificity (95% CI)
2D-XGB	Maximum size; Maximum size of the solid component; CTR	0.766 (0.648-0.869)	1.000 (0.868-1.000)	0.432 (0.271-0.605)
3D-RF	Mass; CTRV_-500_; CTRV_-300_; CTRV_-100_	0.847 (0.745-0.931)	0.731 (0.522-0.884)	0.811 (0.648-0.920)

The final 2D-XGB and 3D-RF algorithms were selected according to their pooled OOF AUCs obtained from nested cross-validation in the training set. Final feature selection and hyperparameter optimization were performed using the complete training set before model refitting. The resulting models were subsequently evaluated once in the held-out test set. Sensitivity and specificity were calculated using the corresponding cutoffs derived from the training-set OOF predictions. OOF, out-of-fold; AUC, area under the receiver operating characteristic curve; CI, confidence interval; RF, random forest; XGB, extreme gradient boosting; CTRV, consolidation-to-tumor ratio of volume at different thresholds.

**Figure 5:**
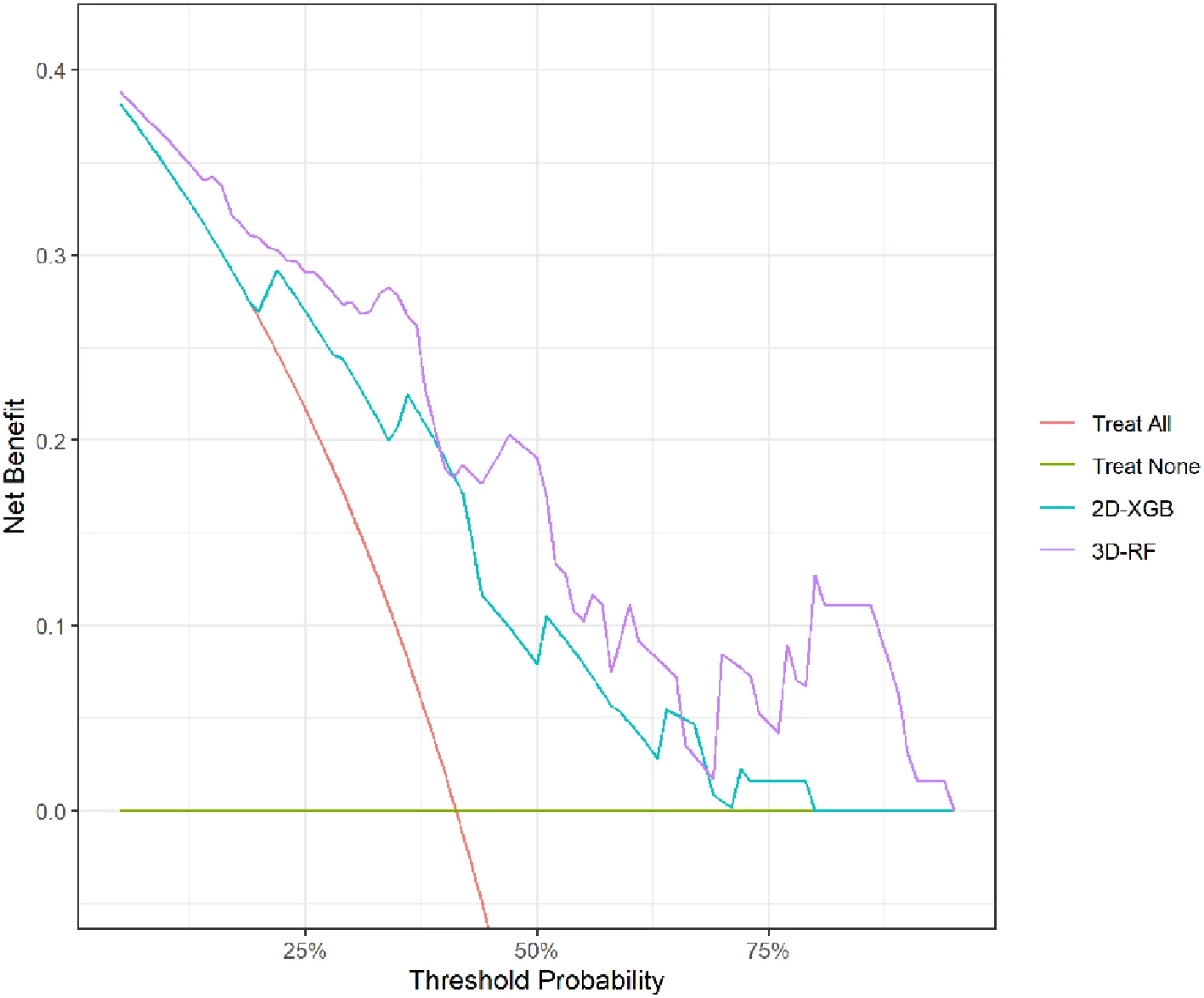
Decision curve analysis of the final 2D-XGB and 3D-RF models in the held-out test set. Net benefit is plotted against threshold probability. The “treat all” and “treat none” strategies are shown as reference approaches. A model provides potential clinical utility when its decision curve yields a higher net benefit than both reference strategies over a given range of threshold probabilities. DCA, decision curve analysis; RF, random forest; XGB, extreme gradient boosting.

**Figure 6:**
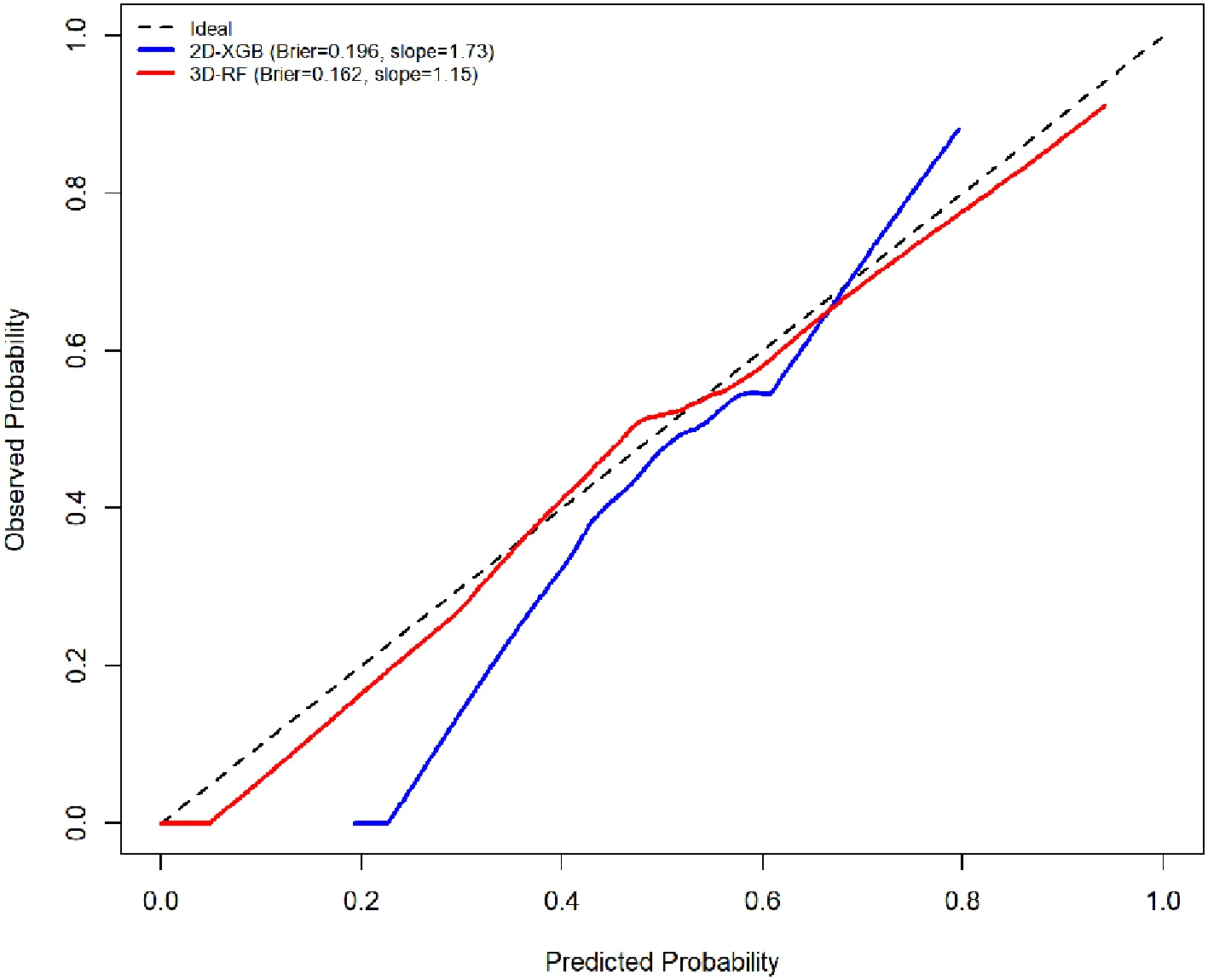
Calibration curves of the final 2D-XGB and 3D-RF models in the held-out test set. Predicted probabilities are plotted against observed event probabilities. The diagonal dashed line represents perfect calibration, whereas the model-specific curves represent the agreement between predicted and observed probabilities for the 2D-XGB and 3D-RF models. RF, random forest; XGB, extreme gradient boosting.

## Discussion

In this cohort, permutation importance analysis of the 2D-XGB model showed that CTR produced the largest decrease in AUC after permutation, indicating that model discrimination was most dependent on this feature among the evaluated predictors. CTR was also selected in all five outer folds during LASSO feature selection, corresponding to a selection frequency of 100% and supporting its consistent selection across folds. Previous studies have shown that CTR reflects the proportion of the solid component within pulmonary nodules and is useful for assessing tumor invasiveness and informing surgical strategies (7–10). Consistent with these findings, our results support the importance of CTR as a 2D imaging parameter for risk stratification, as it remained consistently selected and showed high permutation importance when evaluated alongside multiple imaging features.

In contrast to the 2D-XGB model, feature importance was more distributed across predictors in the 3D-RF model. Mass and CTRV at multiple density thresholds, including CTRV_-100_, CTRV_-500_, and CTRV_-300_, showed notable permutation importance, with fold-wise LASSO selection frequencies of 0.6, 0.6, 0.4, and 0.4, respectively. Among these parameters, mass has been less extensively investigated in clinical studies. By integrating nodule volume and CT attenuation, mass provides a composite measure of lesion size and density and may therefore capture information not represented by either volume or mean CT attenuation alone. Previous studies have suggested that mass may be a useful quantitative parameter for the longitudinal assessment of pulmonary nodules (19, 20). In the present study, the contribution of mass to the 3D-RF model suggests that this parameter may also have potential value for risk stratification, although further validation is required.

Notably, the CT attenuation thresholds used to define the 3D solid component in the present study differed from those used in some previous studies. Nomori et al. defined regions with CT attenuation values ≥−300 HU as solid components based on the peaks of CT attenuation histograms. Two subsequent studies adopted the same threshold and defined regions with attenuation values ranging from −800 to −300 HU as ground-glass components (16, 21, 22). However, other studies have used different thresholds (17, 19, 23). Liu et al. used CTRV_−250_ as the primary threshold, which showed a relatively strong association in univariable analysis; however, semantic features and VSC were not simultaneously incorporated into their model (17). Variability in threshold selection across studies may be related to differences in study populations, grouping strategies, CT acquisition and reconstruction parameters, and image-analysis methods. Collectively, these differences highlight the lack of a standardized CT attenuation threshold for defining the 3D solid component.

Among the CTRV parameters evaluated, CTRV_-100_, CTRV_-300_, and CTRV_-500_ were retained in the final 3D model. Among these, CTRV_-100_ showed the greatest permutation importance, whereas CTRV_-300_ and CTRV_-500_ also contributed to model discrimination. These findings suggest that volumetric information derived from multiple CT attenuation thresholds may capture complementary aspects of the distribution of solid components within nodules. However, because the present study did not directly compare single-threshold and multi-threshold models, the added value of combining multiple thresholds requires further validation. In addition, the feature importance analyses of the 2D and 3D models showed that CTR, mass, and CTRV at different thresholds all reflect aspects of the solid component to varying degrees. Their contribution to the models is consistent with previous studies suggesting that solid component–related parameters are closely associated with the degree of invasion (9, 24, 25).

In addition to quantitative CT parameters, semantic features, including irregularity, spiculation, lobulation, pleural indentation, and air bronchogram, were incorporated into the models. In the 2D-XGB model, air bronchogram, spiculation, and lobulation showed notable permutation importance, whereas all five semantic features contributed to varying degrees in the 3D-RF model. Previous studies have demonstrated associations between several semantic features and the risk of malignancy in pulmonary nodules. Irregular margins have been significantly associated with malignant pulmonary nodules (26–29), while spiculation has been incorporated into several large-sample prediction models for malignancy risk assessment (30–33). Lobulation is also a common imaging feature of malignant nodules (26) and has been included as a predictor in multiple previously developed models (34). Similarly, pleural indentation, also referred to as pleural tagging, has been reported as a common feature of malignant pulmonary nodules (35, 36). These morphological features characterize different aspects of nodule shape and margins and may provide information complementary to quantitative CT parameters. Although previous studies have primarily examined their associations with malignancy, our findings suggest that some of these features may also contribute to distinguishing the degree of invasion among malignant SSNs.

Compared with the classic morphological features discussed above, air bronchogram has received increasing attention in pulmonary nodule risk assessment, with recent studies reporting an association with malignant pulmonary nodules (37, 38). In the present study, air bronchogram ranked third in the permutation importance analyses of both the 2D-XGB and 3D-RF models and showed relatively high importance among the included semantic features. This finding suggests that air bronchogram may contain imaging information relevant not only to malignancy but also to the degree of invasion. One possible explanation is that air bronchogram may reflect tumor growth along alveolar structures and changes in residual or involved bronchi within the lesion, thereby providing information on the internal architecture of the nodule. In contrast to features such as spiculation and lobulation, which primarily describe external morphology, air bronchogram may therefore capture a different aspect of nodule characteristics. However, semantic feature assessment is inherently subjective, and the present findings do not establish that air bronchogram is superior to other conventional semantic features. Its potential value for risk stratification requires further validation in larger populations and independent external cohorts.

The present study compared the 2D-XGB and 3D-RF models in terms of discrimination, calibration, and clinical net benefit. The paired DeLong test showed no significant difference in AUC between the two models based on either the pooled OOF predictions or the held-out test set predictions, indicating comparable discriminative performance. However, AUC reflects only a model’s ability to distinguish between individuals with different outcome risks and does not fully capture the accuracy of predicted probabilities or potential clinical utility.

DCA showed that both 2D-XGB and 3D-RF provided net benefit across a broad range of threshold probabilities in the held-out test set. Differences between the two models became more apparent as the threshold probability increased. The net benefit of 2D-XGB approached zero at threshold probabilities above approximately 70%, whereas 3D-RF maintained positive net benefit over a broader threshold range and generally showed higher net benefit than 2D-XGB at intermediate-to-high threshold probabilities. These findings suggest that, despite comparable discriminative performance, 3D-RF may provide potential clinical benefit across a broader range of decision thresholds. The 3D-RF model also showed better calibration than the 2D-XGB model in the held-out test set. Its calibration curve was generally closer to the ideal reference line, with a lower Brier score and a calibration slope closer to 1. These findings indicate closer agreement between the predicted probabilities and observed outcomes for 3D-RF. Taken together, although 3D-RF did not show significantly better discrimination than 2D-XGB, it demonstrated better calibration and provided net benefit across a broader range of threshold probabilities in the held-out test set.

In this study, AI software was used for fully automated quantification of 3D CT parameters. Previous studies have demonstrated the reliability of semi-automated and automated measurements and their potential to reduce interobserver variability (13, 39, 40). However, current AI-based segmentation may not always accurately distinguish bronchi and blood vessels traversing a nodule, which may introduce errors in attenuation-based measurements (23, 41). AI-based tools are increasingly being applied to pulmonary nodule assessment, including prediction of invasion and three-dimensional reconstruction of pulmonary vessels and bronchi for segmentectomy planning (42–44). Further improvements in segmentation and structure recognition may enhance the accuracy and standardization of automated 3D quantitative assessment.

The present study showed that the 3D-based model did not achieve significantly better discrimination than the 2D-based model but demonstrated better calibration and a broader range of net benefit on DCA. These findings suggest that 3D parameters may provide additional information for risk probability estimation and potentially support clinical decision-making. In recent years, various 3D imaging parameters have increasingly been applied to pulmonary nodule assessment. Some studies have also evaluated nodule invasiveness using the maximum standardized uptake value derived from F-18 fluorodeoxyglucose-positron emission tomography/CT (22, 23, 45). However, PET/CT is relatively expensive and may not be suitable for all patients. Moreover, standardized uptake value measurements may vary according to imaging equipment, acquisition protocols, and quantitative methods across centers, potentially limiting their reproducibility and broader clinical application.

In comparison, 3D quantitative parameters derived from conventional chest CT may have broader clinical applicability. Nodule volume has been incorporated into guidelines for pulmonary nodule follow-up and growth assessment (24, 46), and mean CT attenuation has also been reported to be associated with the degree of invasion of pulmonary nodules (41, 45). In the present study, however, neither volume nor mean CT attenuation was retained in the final 3D model, whereas mass, which integrates information on both lesion volume and CT attenuation, showed greater permutation importance. This finding suggests that mass may capture complementary information related to both lesion size and density. Although evidence regarding mass remains relatively limited, the present results support its potential value as a 3D quantitative parameter for assessing the degree of invasion and for risk stratification, warranting further validation.

In addition, the present findings suggest that defining the solid component using a single CT attenuation threshold may not fully capture the imaging characteristics associated with nodule invasiveness. Although the multiple CTRV parameters retained in the 3D model inevitably contained overlapping information, each showed non-negligible permutation importance. This finding raises the possibility that a single optimal threshold may not fully characterize solid components associated with invasiveness and that relevant information may instead be distributed across multiple attenuation ranges. CTRV values derived at different thresholds may therefore provide complementary information on the distribution of solid components within a nodule. However, because the present study did not directly compare single-threshold and multi-threshold models, whether combining multiple thresholds provides incremental predictive value cannot be determined from the current results. Further studies in larger samples and independent external cohorts are needed to determine whether multi-threshold combinations can consistently improve model performance.

From a measurement perspective, 3D parameters may partly address some limitations of conventional 2D measurements. CTR is calculated as the ratio of the maximum diameter of the solid component to the maximum diameter of the nodule. In PSNs with scattered or irregular solid components, a single linear measurement may not fully represent the overall extent of the solid component. In contrast, CTRV is derived from the entire nodule volume and may reduce the dependence on measurement-plane selection while providing a more comprehensive assessment of the spatial distribution of solid components within the lesion. Previous studies have also used the maximum diameter of the solid component as an important criterion for T staging of lung adenocarcinoma (24, 25). Although the VSC was not retained in the final 3D model in the present study, its potential biological and clinical relevance warrants further investigation.

Furthermore, CTR has been used to support surgical decision-making for pulmonary nodules, and the predictive contribution of CTRV observed in the present study suggests that 3D assessment of the solid component may also have potential value in surgical planning. This may be particularly relevant for PSNs in which the solid component is difficult to characterize adequately using CTR alone, as CTRV provides a volumetric assessment of the solid component. However, the present study did not evaluate surgical outcomes, recurrence, or survival. Future prospective, multicenter studies with larger sample sizes and long-term follow-up are therefore needed to clarify the relationships between CTRV at different attenuation thresholds and postoperative recurrence and survival outcomes, and to determine whether CTRV can contribute to individualized surgical decision-making.

This study has several limitations. First, this was a single-center retrospective study with a relatively limited sample size, and the models were evaluated only in a held-out test set. Therefore, their generalizability remains to be established through external validation in larger, multicenter cohorts. Second, model development was based primarily on clinical characteristics and CT-derived imaging features; other potentially relevant information, such as laboratory data, was not incorporated. Future studies integrating additional clinical variables may enable a more comprehensive assessment. Third, CT images were acquired using different scanners, which may have introduced variability in quantitative imaging measurements. Although standardized acquisition and reconstruction parameters were used in this study, further investigation is needed to assess the robustness of 3D quantitative parameters across different scanners and imaging protocols.

## Data Availability

The raw data supporting the conclusions of this article will be made available by the authors, without undue reservation.

## Supplementary material

Supplementary Figure 1 LASSO feature selection for the final 2D and 3D models using the complete training set. **(A)** LASSO coefficient profiles of candidate continuous imaging features for the 2D model. **(B)** LASSO coefficient profiles of candidate continuous imaging features for the 3D model. **(C)** Cross-validated binomial deviance for selection of the regularization parameter λ in the 2D model. **(D)** Cross-validated binomial deviance for selection of λ in the 3D model. The optimal λ was selected according to the minimum-deviance criterion (λ min), and features with nonzero coefficients at λ min were retained. LASSO, least absolute shrinkage and selection operator.
