# Supplementary material for: Comparative analysis of two-dimensional and three-dimensional machine learning models for predicting the degree of invasion in lung non-mucinous adenocarcinomas presenting as subsolid nodules
Source: Front Oncol. 2026 Sep 16;16:1916349. doi: 10.3389/fonc.2026.1916349 (PMC13623560; doi:10.3389/fonc.2026.1916349)
Supplement: Supplementary file 1 [file Table1.docx]

All machine learning analyses were performed using R version 4.6.1; specific R packages and their corresponding versions are listed in Table S1.

| **Table S1** Software packages and versions | |
| --- | --- |
| Package | Version |
| readxl | 1.5.0 |
| caret | 7.0-1 |
| glmnet | 5 |
| e1071 | 1.7-17 |
| randomForest | 4.7-1.2 |
| xgboost | 3.2.1.1 |
| lightgbm | 4.7.0 |
| pROC | 1.19.0.1 |
| dcurves | 0.5.1 |
| ggplot2 | 4.0.3 |

Prior to model building, outcome variables and binary variables were encoded as 0/1, and continuous variables were converted to numerical format. No global normalization or standardization was performed prior to cross-validation. For LASSO regression, continuous predictor variables were internally standardized by `glmnet` within the corresponding training subsets (`standardize = TRUE`). For SVM, feature scaling was performed internally based on the corresponding training data (`scale = TRUE`). No additional global scaling was performed for logistic regression (LR), random forest (RF), XGBoost, or LightGBM models.

There were no missing values in the raw data; therefore, missing value imputation was neither required nor performed.

To ensure reproducibility, a prespecified master random seed of 777 was used. Deterministic seed offsets derived from the master seed were used throughout the cross-validation, feature selection, hyperparameter tuning, and model fitting processes. Model development and internal validation employed a nested cross-validation framework consisting of 5 outer folds × 5 inner folds. Stratified partitioning based on the outcome variable was used to approximately preserve the class distribution within each fold. SMOTE, oversampling, undersampling, or class weighting were not applied.

The complete prespecified hyperparameter search grids for LR, SVM, RF, XGBoost, and LightGBM are provided in Table S2.

| **Table S2** Prespecified hyperparameter search grids | | |
| --- | --- | --- |
| Model | Hyperparameter | Search range |
| LR | - | No hyperparameter tuning |
| Linear SVM | Cost | 0.1, 1, 10 |
| RF | mtry | 1, 2, 3, 5 |
| RF | nodesize | 1, 5 |
| RF | ntree | 500, fixed |
| XGBoost | max depth | 2, 4 |
| XGBoost | learning rate | 0.05, 0.10 |
| XGBoost | nrounds | 50, 100 |
| LightGBM | num leaves | 7, 15 |
| LightGBM | learning rate | 0.03, 0.05 |
| LightGBM | nrounds | 50, 100 |
| LASSO | alpha | 1, fixed |
| LASSO | λ | lambda.min, determined by CV |
| LASSO | CV metric | Binomial deviance |
